# Simultaneous measurements of tissue blood flow and oxygenation using a wearable fiber-free optical sensor

**DOI:** 10.1117/1.JBO.26.1.012705

**Published:** 2021-01-29

**Authors:** Xuhui Liu, Yutong Gu, Chong Huang, Mingjun Zhao, Yanda Cheng, Elie G. Abu Jawdeh, Henrietta S. Bada, Lei Chen, Guoqiang Yu

**Affiliations:** a University of Kentucky, Department of Biomedical Engineering, Lexington, Kentucky, United States; b University of Kentucky, Department of Pediatrics, College of Medicine, Lexington, Kentucky, United States; c University of Kentucky, Department of Physiology, Spinal Cord and Brain Injury Research Center, Lexington, Kentucky, United States

**Keywords:** speckle contrast, blood flow, blood oxygenation, wearable, deep tissue

## Abstract

**Significance:** There is an essential need to develop wearable multimodality technologies that can continuously measure both blood flow and oxygenation in deep tissues to investigate and manage various vascular/cellular diseases.

**Aim:** To develop a wearable dual-wavelength diffuse speckle contrast flow oximetry (DSCFO) for simultaneous measurements of blood flow and oxygenation variations in deep tissues.

**Approach:** A wearable fiber-free DSCFO probe was fabricated using 3D printing to confine two small near-infrared laser diodes and a tiny CMOS camera in positions for DSCFO measurements. The spatial diffuse speckle contrast and light intensity measurements at the two different wavelengths enable quantification of tissue blood flow and oxygenation, respectively. The DSCFO was first calibrated using tissue phantoms and then tested in adult forearms during artery cuff occlusion.

**Results:** Phantom tests determined the largest effective source–detector distance (15 mm) and optimal camera exposure time (10 ms) and verified the accuracy of DSCFO in measuring absorption coefficient variations. The DSCFO detected substantial changes in forearm blood flow and oxygenation resulting from the artery occlusion, which meet physiological expectations and are consistent with previous study results.

**Conclusions:** The wearable DSCFO may be used for continuous and simultaneous monitoring of blood flow and oxygenation variations in freely behaving subjects.

## 1 Introduction

Oxygen is a critical component of the microenvironment required for supporting cellular activities. Tissue oxygen alteration is a vital sign for the assessment of cardiovascular diseases such as heart failure, septic shock, and cerebral hypoxia.^1^^–^^3^ Tissue oxygenation level reflects the balance between oxygen supply and consumption.^4^ Blood flow affects the efficiency of oxygen delivery to and waste removal from tissues. Blood flow is highly sensitive to pathophysiological alterations,^5^ thus could be an indicator for the detection of diseases that are associated with tissue ischemia such as peripheral artery disease, cerebral vascular disease, neurological disorders, and cancers.^6^^–^^11^ There are also growing interests in simultaneous measurements of blood flow and oxygenation alterations as more comprehensive biomarkers for tissue health/injury than one single parameter alone.^12^^–^^14^ Furthermore, a combination of tissue blood flow and oxygenation allows to estimate the metabolic rate of tissue oxygen consumption,^12^^,^^15^ another important functional parameter highly associated with tissue pathology.

Measurements of deep tissue hemodynamics provide critical information for diagnosis and therapeutic assessment of diseases affecting large tissue volumes, such as large tumors,^16^ deep tissue burns/wounds,^17^ and brain tissue injuries.^18^^,^^19^ For instance, a deep penetration is necessary for noninvasive transcranial measurements of cerebral blood flow and oxygenation through intact scalp and skull. Moreover, wearable technologies enable continuous and longitudinal monitoring of tissue hemodynamics in conscious, freely behaving subjects, thus advancing our understanding of cognitive processes and adaptive behavior.^20^^,^^21^ Therefore, there is an urgent need to develop wearable, multimodality technologies that can noninvasively measure both blood flow and oxygenation in deep tissues to investigate pathologies and interventions for various vascular/cellular diseases.

A variety of technologies have been developed for measurements of tissue hemodynamics. In contrast to large imaging modalities such as magnetic resonance imaging, computed tomography, and positron emission tomography, optical instruments are fast, continuous, portable, and inexpensive, and have the potential to become wearable tools. For example, wearable pulse oximeters have been extensively used in clinics for continuous monitoring of blood oxygen saturation (${\mathrm{SaO}}_{2}$) levels in peripheral arteries.^22^^,^^23^ However, global ${\mathrm{SaO}}_{2}$ is not always consistent with tissue blood oxygen saturation (${\mathrm{StO}}_{2}$) in a specific organ/region (e.g., brain).

Near-infrared diffuse optical techniques are broadly used to probe deep tissue properties (up to several centimeters), including near-infrared spectroscopy (NIRS) for tissue oxygenation measurements and diffuse correlation spectroscopy (DCS) for tissue blood flow measurements.^24^^–^^29^ Conventional NIRS measures light intensity attenuations by tissue absorption and scattering at multiple wavelengths to calculate changes in oxy- and deoxy-hemoglobin concentrations ($\left[{\mathrm{HbO}}_{2}\right]$ and [Hb]). A variety of wearable NIRS techniques have been developed and made significant contributions to neuroscience,^30^^–^^35^ although most systems require heavy instrumentation and cable bundles between the device and probe, which encumber the subject and may cause the subject’s discomfort. More recently, a few miniaturized and/or wireless NIRS systems significantly reduce device dimension/weight and comfort subjects.^36^^–^^40^ The relatively new DCS systems detect temporal diffuse laser speckle fluctuations resulting from red blood cell motions in the microvasculature.^9^^,^^13^^,^^17^^,^^24^^–^^29^^,^^41^^,^^42^ Although effective, DCS utilizes large and expensive long-coherence lasers as sources and single-photon-counting avalanche photodiodes as detectors, which cannot be directly placed on target tissues. Thus, rigid and fragile optical fibers are usually used for source and detector couplings, which significantly constrain the subject’s movement.

We have previously developed and tested an innovative, wearable, single-wavelength diffuse speckle contrast flowmetry (DSCF) technique, which provides a simple, low-cost, fiber-free, and compact method for continuous monitoring of blood flow variations in deep tissues of animals and humans.^21^^,^^43^ The DSCF uses a small laser diode as a focused-point source for deep tissue penetration and a tiny CMOS camera as a high-density two-dimensional (2D) detector array to detect spontaneous spatial fluctuations of diffuse laser speckles resulting from movement of red blood cells (i.e., blood flow). Thousands of pixels on the CMOS sensor significantly improved the sampling rate/density and reduced the cost and dimension of the probe/device. Importantly, the connections between the DSCF probe and a control unit are all flexible electrical wires (i.e., fiber-free), making it possible to build a wearable system for continuous monitoring of tissue blood flow variations in freely behaving subjects.^21^^,^^43^

The goal of this study was to extend the innovative DSCF device to a dual-wavelength diffuse speckle contrast flow oximetry (DSCFO) system for simultaneous measurements of blood flow and oxygenation variations in deep tissues. A wearable DSCFO probe was fabricated using 3D printing to confine two small laser diodes (at different wavelengths) and a tiny NanEye camera in positions for DSCFO measurements. The spatial diffuse speckle contrast and light intensity measurements at the two wavelengths enable quantification of tissue blood flow and oxygenation, respectively. A printed circuit board was designed to control and monitor the output powers of laser diodes via serial interface. A graphical user interface (GUI) was developed to control the DSCFO device and display blood flow and oxygenation variations in real time. The DSCFO system was first tested and calibrated against a commercial NIRS device (Imagent, ISS) in standard tissue phantoms with known optical properties. The feasibility of the DSCFO for *in-vivo* measurements was then verified by simultaneous measurements of forearm blood flow and oxygenation variations during artery cuff occlusions on the upper arms in healthy adults.

## 2 Methods and Materials

### 2.1 Innovative Diffuse Speckle Contrast Flow Oximetry (DSCFO)

#### 2.1.1 Dual-wavelength DSCFO system

**Fig. 1:**
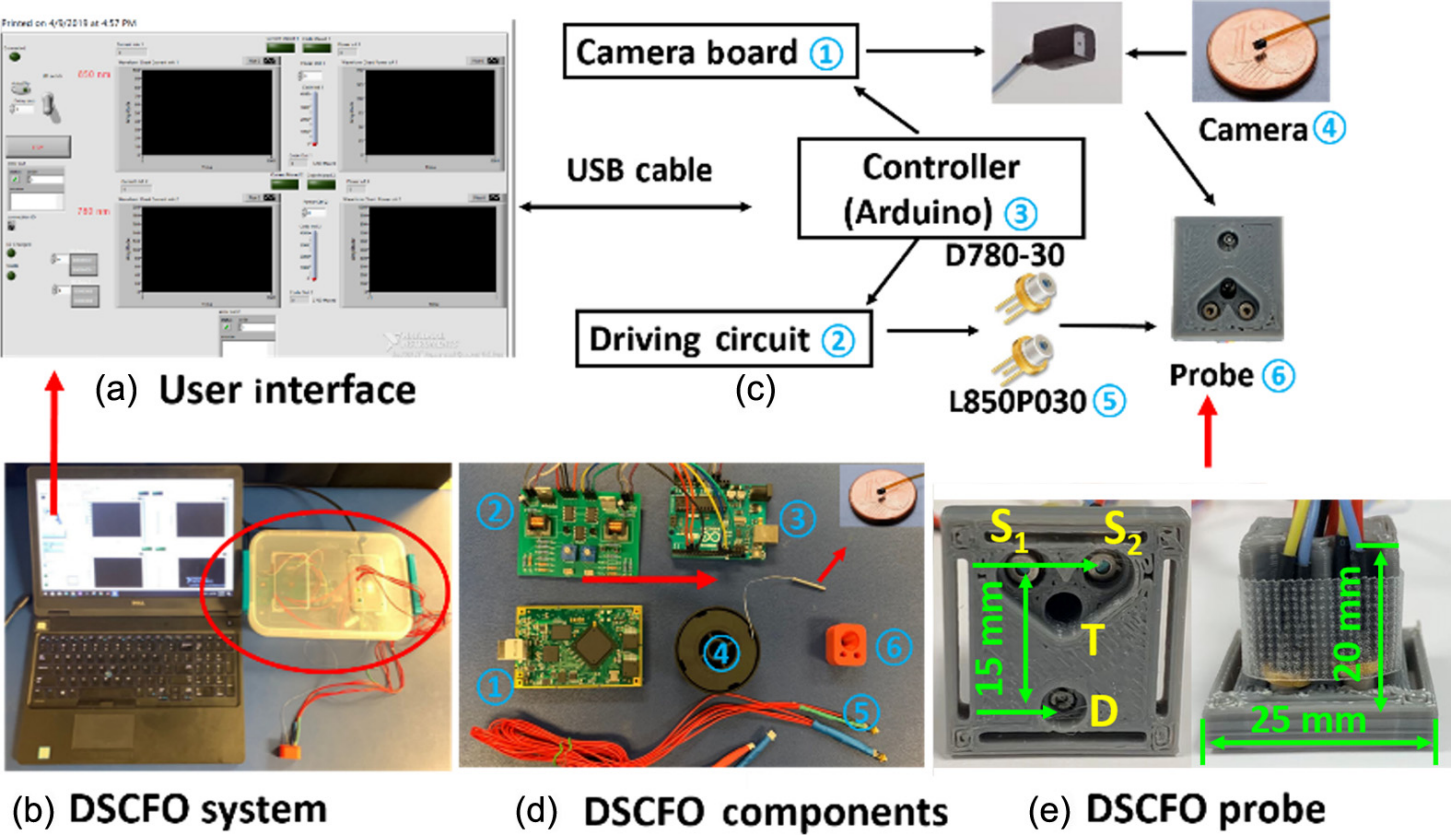
Dual-wavelength DSCFO system for deep tissue blood flow and oxygenation measurements. (a) The GUI of the DSCFO device. (b) The DSCFO system, including a laptop and the DSCFO device. (c) A schematic of DSCFO device. (d) Components in the DSCFO device including camera electronic (NanEye USB 2.0) ①, laser diodes driving circuit ②, Arduino controller ③, NanEye camera ④, laser diodes ⑤, and DSCFO probe ⑥. (e) The wearable 3D-printed DSCFO probe. Two laser diodes ($S_{1}$: 780 nm, 30 mW and $S_{2}$: 850 nm, 30 mW) work as the dual-wavelength sources. The NanEye camera (D) works as a 2D detector array. The thermistor resistor (T) works as a thermal sensor to detect skin temperature. The probe dimensions are $25\times 25\times 20\;{\mathrm{mm}}^{3}$. The S-D distances are 15 mm.

We designed a dual-channel circuit board (driving two laser diodes) ② that packed with a camera electronic board ① and an Arduino controller ③ to form a low-cost, compact, dual-wavelength DSCFO device (Fig. 1). Communication between the device and a user laptop goes through a universal serial bus (USB) cable. An ultra-small low-power camera (NanEye 2D Black and White; dimension: $1\times 1\;{\mathrm{mm}}^{2}$, power: 4 mW, Awaiba) was used as a 2D detector to provide a 250×250  pixel-array (pixel dimensions: $3\times 3\;{\mu m}^{2}$) ④. A tiny optical lens with a focus length of less than 1 mm was integrated on top of the camera sensor chip (dimensions: $0.75\times 0.75\;{\mathrm{mm}}^{2}$; F number: 4). The dynamic range of NanEye sensor is 58 dB. Two small laser diodes ⑤ (D780-30, Ø5.6 mm, 30 mW, 780 nm, US-Lasers; L850P030, Ø5.6  mm, 30 mW, 850 nm, Thorlabs) were controlled by a customized feedback circuit to generate constant light intensity outputs, as was done in our previous DSCF device.^21^ Briefly, a built-in photodiode in the laser diode package continuously detected the light intensity generated by the laser diode. The feedback circuit controlled the driving current to the laser diode to stabilize light intensity output. The two laser diodes ⑤ and NanEye camera ④ were fixed to a small wearable probe ⑥ and connected to the compact device through flexible electrical wires (i.e., fiber-free). A GUI (Microsoft C#) was developed to control the camera and display the results in real time. A LabVIEW™ (National Instruments) program was designed to monitor and control the light intensity outputs of laser diodes. The synchronization between the laser diodes and camera was realized via the LabVIEW program in the control panel (laptop).

#### 2.1.2 Novel wearable fiber-free DSCFO probe

DSCFO allows continuous measurements of blood flow and oxygenation in deep tissue via a wearable miniaturized probe tightly attached on the tissue surface using medical tapes (Transpore, 3M) [Fig. 1(e)]. The DSCFO probe was designed using SOLIDWORKS (Dassault Systemes) and fabricated by a 3D printer (X-Max, Qidi Tech) with nontransparent polylactic acid materials. For protection, the NanEye camera was wrapped with a thin transparent film and inserted into a transparent plastic tube. The camera with the protection tube was then installed into the probe and the working distance between the integrated camera lens and tissue surface was ∼3  mm to ensure appropriate optical focus. The NanEye camera imaged a field-of-view (FOV) of $3\times 3\;{\mathrm{mm}}^{2}$ at this working distance. The bodies of the installed laser diodes were exposed to the air, allowing for sufficient air circulation to spread the heat generated by the laser diodes. The source–detector (S-D) distances between the two laser diodes and the camera were set as 15 mm, allowing a maximal penetration depth of ˜8  mm.^26^^,^^44^^–^^46^ Importantly, connections between the probe and the device were all soft and flexible electrical cables/wires (i.e., fiber-free), allowing for the use in conscious, freely moving subjects. For safety, a tiny thermistor (Allied Electronics and Automation) calibrated against a thermometer (AcuRite) was installed close to the two laser diodes to continuously monitor temperature on the skin surface.

#### 2.1.3 DSCFO data analyses

**Fig. 2:**
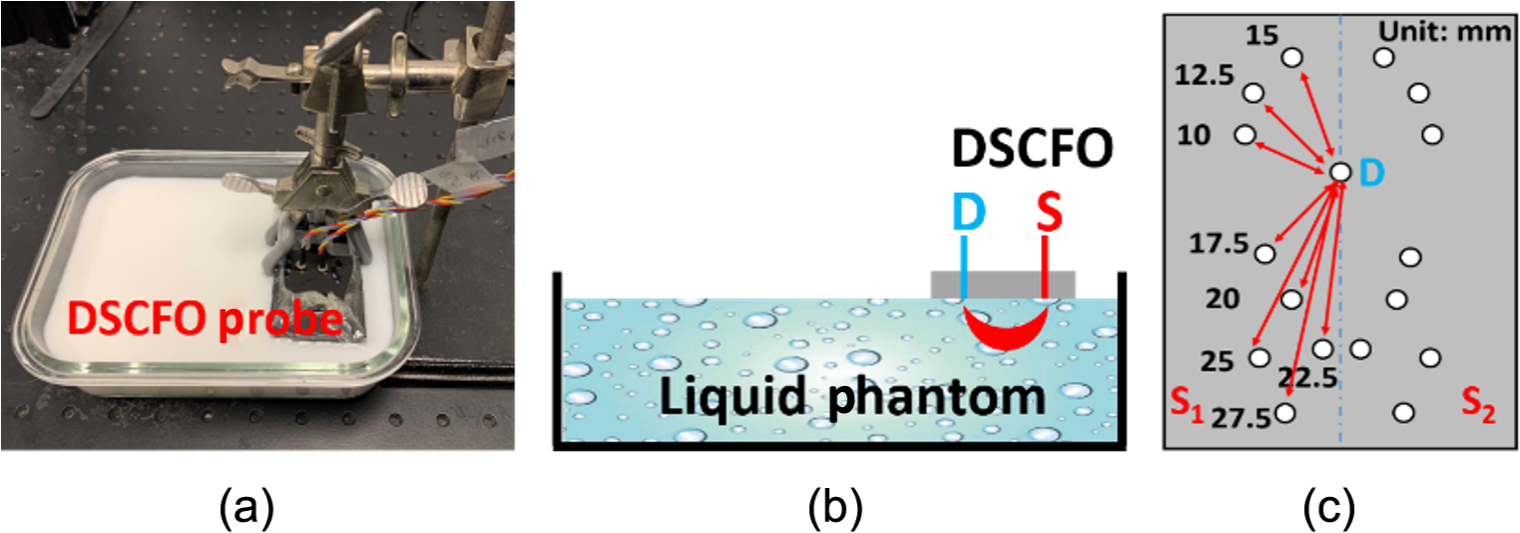
Experimental setup for testing the sensitivities and SNRs of DSCFO measurements using an Intralipid tissue phantom. (a) A DSCFO probe was placed on the surface of the Intralipid liquid phantom by a mechanical holder. (b) A NanEye camera (D) and two laser diodes ($S_{1}=780\;\mathrm{nm}$ and $S_{2}=850\;\mathrm{nm}$) were confined by a black foam pad to form a DSCFO probe. (c) The S-D configuration on the DSCFO probe.

**Fig. 3:**
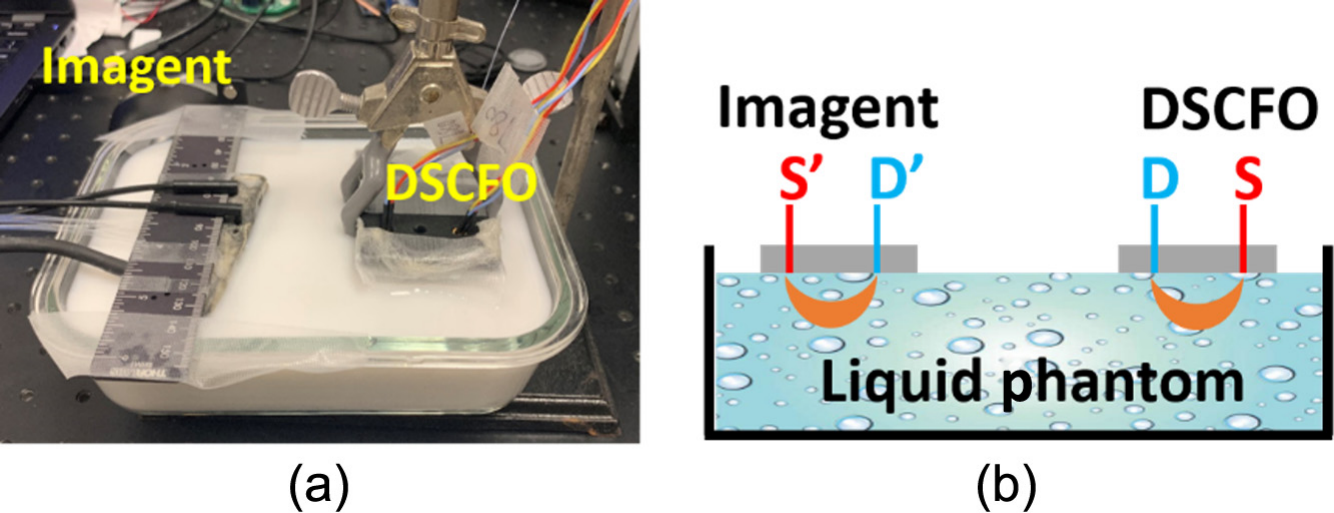
Experimental setup for concurrent DSCFO and Imagent measurements of $\Delta \mu_{a}$ during ink titration. The DSCFO and Imagent probes were placed on the surface of the liquid phantom in an aquarium. The S-D distances for DSCFO and Imagent measurements were 15 and 20 mm, respectively.

The DSCFO alternatively generates raw intensity images at two wavelengths with an exposure time (T) on the scale of milliseconds. Our previous studies have described the methods for extracting blood flow information from single-wavelength DSCF measurements.^21^^,^^43^ Briefly, photons generated by near-infrared laser diode diffuse through the tissue volume via a “banana-shape” pathway (see Figs. 2 and 3). The NanEye camera captures spatial laser speckle contrasts on tissue surface caused by movement of red blood cells in the measured tissue volume. The laser speckle size with the S-D configuration in DSCFO is estimated by Eq. (1)^47^

$$\rho_{\text{speckle}}=2.44\lambda (1+M)/\#,$$ (1)

where λ is the wavelength of light and M and f/# are the magnification and F number of the imaging system, respectively. Based on the specifications of NanEye camera (pixel size: $\rho_{\text{pixel}}=3\;\mu m$; image size: $\rho_{\text{image}}=0.75\;\mathrm{mm}$; object size: $\rho_{\text{object}}=3\;\mathrm{mm}$; F number: f/#=4), the magnification M is 0.25 ($\rho_{\text{image}}/\rho_{\text{object}}$) at the working distance of ∼3  mm. Thus, the laser speckle size ($\rho_{\text{speckle}}=9.5\;\mu m$) satisfies the Nyquist sampling criterion, $\rho_{\text{speckle}}>2\rho_{\text{pixel}}$.^47^

A window of 7×7  pixels is commonly used to quantify the spatial laser speckle contrast ($K_{s}$) in conventional laser speckle contrast imaging and our DSCF measurements.^21^^,^^43^^,^^47^ $K_{s}$ is calculated by the ratio of standard deviation (σ) and mean (μ) over the selected window of 49 pixels (i.e., $K_{s}=\sigma /\mu$).

Preprocessing of raw images is required and described in our previous publications.^21^^,^^43^^,^^48^ Briefly, the laser speckle contrast after shot and dark noise corrections is given by Eq. (2)

$$K_{s}=\sqrt{\frac{\sigma^{2}(I)-\sigma^{2}(I_{D})-\sigma_{S}^{2}(I_{C})}{\mu^{2}(I_{C})}},$$ (2)

where I is the light intensity of a single pixel and $I_{D}$ is the intensity of dark current. The shot noise ($I_{C}=I-I_{D}$) follows Poisson statistics: $\sigma_{s}(I_{C})=\sqrt{\mu (I_{C})}$, which is incorporated into the correction calculation [Eq. (2)].

To increase the signal-to-noise ratio (SNR), a 5×5 adjacent pixel window (7×7  pixels in each window) with 25 values of $K_{s}$ at the center of the camera sensor was averaged, representing a single detector with a detection area of $∼0.18\;{\mathrm{mm}}^{2}$. A blood flow index (BFI) was then extracted through a nonlinear relationship between the $K_{s}$ and BFI under a semi-infinite geometry.^43^^,^^49^ The relative change in BFI (rBF) was calculated by normalizing BFI data to the baseline value before physiological changes.

Tissue blood oxygenation variation was extracted from the measured light intensities at two wavelengths based on the modified Beer–Lambert law [Eqs. (3)–(5)]^50^

$$\Delta \mu_{a}(\lambda )=\ln(\frac{I_{\lambda B}}{I_{\lambda T}})/\rho {\mathrm{DPF}}_{\lambda },$$ (3)

$$\Delta [{\mathrm{HbO}}_{2}]=\frac{\varepsilon_{\mathrm{Hb}}(\lambda_{1})\Delta \mu_{a}(\lambda_{2})-\varepsilon_{\mathrm{Hb}}(\lambda_{2})\Delta \mu_{a}(\lambda_{1})}{\varepsilon_{\mathrm{Hb}}(\lambda_{1})\varepsilon_{{\mathrm{HbO}}_{2}}(\lambda_{2})-\varepsilon_{{\mathrm{HbO}}_{2}}(\lambda_{1})\varepsilon_{\mathrm{Hb}}(\lambda_{2})},$$ (4)

$$\Delta [\mathrm{Hb}]=\;\frac{\varepsilon_{{\mathrm{HbO}}_{2}}(\lambda_{2})\Delta \mu_{a}(\lambda_{1})-\varepsilon_{{\mathrm{HbO}}_{2}}(\lambda_{1})\Delta \mu_{a}(\lambda_{2})}{\varepsilon_{\mathrm{Hb}}(\lambda_{1})\varepsilon_{{\mathrm{HbO}}_{2}}(\lambda_{2})-\varepsilon_{{\mathrm{HbO}}_{2}}(\lambda_{1})\varepsilon_{\mathrm{Hb}}(\lambda_{2})},$$ (5)

where $\Delta \mu_{a}(\lambda )$ is the relative change of absorption coefficient $\mu_{a}$ at wavelength λ ($\lambda_{1}=780\;\mathrm{nm}$ and $\lambda_{2}=850\;\mathrm{nm}$). The $\varepsilon_{\mathrm{Hb}}(\lambda )$ and $\varepsilon_{{\mathrm{HbO}}_{2}}(\lambda )$ are the extinction coefficients of Hb and ${\mathrm{HbO}}_{2}$.^51^ The $I_{\lambda B}$ and $I_{\lambda T}$ are the measured light intensities at the baseline and at time T, respectively. The differential path factor (${\mathrm{DPF}}_{\lambda }$) is the ratio of the mean photon path length over the S-D distance (ρ), obtained from the literature.^52^^–^^54^

### 2.2 Experimental Protocols

#### 2.2.1 Assessment of measurement sensitivities and SNRs using a standard tissue phantom

The SNR of DSCFO measurement depends on the light intensity detected, which decays with the increase of S-D distance and decrease of camera exposure time (Fig. 2). A DSCFO probe with multiple S-D pairs was designed to test measurement sensitivities and SNRs with varied S-D distances and exposure times. The tiny NanEye camera imaged a small FOV of $3\times 3\;{\mathrm{mm}}^{2}$ on the tissue surface [Fig. 2(c)]. The camera was fixed at one location (D) as a single sensor while the sources (S) were moved to different locations with desired S-D distances. The S-D distance varied from 10 to 27.5 mm with an interval of 2.5 mm [via changing source locations, Fig. 2(c)] while the exposure time (T) changed from 1 to 20 ms with an interval of 5 ms. The probe was placed on the surface of the Intralipid liquid phantom in an aquarium [dimensions: $200\times 170\times 60\;{\mathrm{mm}}^{3}$, Figs. 2(a) and 2(b)]. The liquid phantom, consisting of Intralipid particles, India ink, and distilled water, are commonly used to calibrate NIRS/DCS instruments.^44^^,^^55^^,^^56^ The Intralipid (Fresenius Kabi) was used to provide the particle Brownian motion (flow) and control the reduced scattering coefficient (${\mu_{s}}^{\prime }$) while the India ink (black India) was used to adjust the absorption coefficient $\mu_{a}$. The optical properties of the Intralipid phantom were set as $\mu_{a}=0.025\;{\mathrm{cm}}^{-1}$ and ${\mu_{s}}^{\prime }=8\;{\mathrm{cm}}^{-1}$ at 830 nm to mimic biological tissue. Each measurement with a certain exposure time and S-D distance was performed for 5 s with a sampling rate of 2 Hz at each wavelength for temporal averaging. A stabilization time of 30 s was utilized between data acquisitions across ink titration steps to ensure measurement stability. Room light was turned off during measurements.

#### 2.2.2 Concurrent DSCFO and Imagent measurements of $\Delta \mu_{a}$ during ink titrations in tissue phantoms

Ink titration is commonly used to create $\mu_{a}$ variations ($\Delta \mu_{a}$) in tissue phantoms (Fig. 3). In this study, $\mu_{a}$ varied from 0.04 to $0.16\;{\mathrm{cm}}^{-1}$ at 830 nm with a step interval of $0.02\;{\mathrm{cm}}^{-1}$ via adding India ink. Based on SNR testing results (Fig. 5), camera exposure time was set as 10 ms and S-D distance was set as 15 mm. For validation, DSCFO measurements were compared against a commercial NIRS device (Imagent, ISS).^57^ Imagent is a frequency-domain device working at two wavelengths (690 and 830 nm) and multiple S-D distances (20, 25, 30, and 35 mm), thus enabling measurements of absolute values of $\mu_{a}$ and ${\mu_{s}}^{\prime }$. The distance between DSCFO and Imagent probes was set as 100 mm to prevent interference across the two measurements. The S-D distances for DSCFO and Imagent measurements were 15 and 20 mm, respectively. At each titration step, 20 data points (10 for each wavelength) were collected over 5 s by the DSCFO probe at a sampling rate of 2 Hz. During measurements, room light was turned off and room temperature was controlled (∼25°C) to maintain a stable Intralipid particle flow.

#### 2.2.3 DSCFO measurements of forearm blood flow and oxygenation variations during artery cuff occlusion

**Fig. 4:**
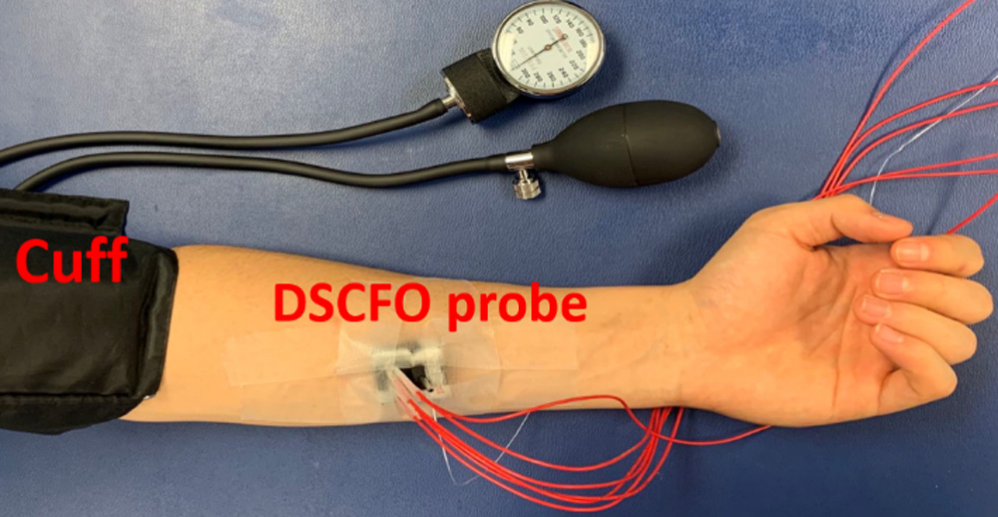
DSCFO measurements of Δ[Hb], $\Delta [{\mathrm{HbO}}_{2}]$, and rBF in the forearm during artery cuff occlusion on the upper arm. A wearable DSCFO probe was fixed on the forearm using medical tapes. An inflatable cuff was installed on the upper arm for artery occlusion.

With approved consent by the University of Kentucky Institutional Review Board, five healthy adults (three males and two females) were recruited for *in-vivo* tests (Fig. 4). The subject sat on a chair to extend his/her arm on a table. A DSCFO probe was taped on the forearm and a cuff was fixed on the upper arm. The DSCFO measurement was continuously performed in each subject for 12 min, including a 2-min baseline, a 5-min artery cuff occlusion (220 mmHg), and a 5-min recovery period. The camera exposure time was 10 ms and S-D distance was 15 mm. Individual DSCFO data were normalized to the mean value of entire baseline measurements over 2 min to calculate relative changes in tissue hemodynamics (Δ[Hb], $\Delta [{\mathrm{HbO}}_{2}]$, and rBF).

## 3 Results

### 3.1 Tissue Phantom Measurement Results

**Fig. 5:**
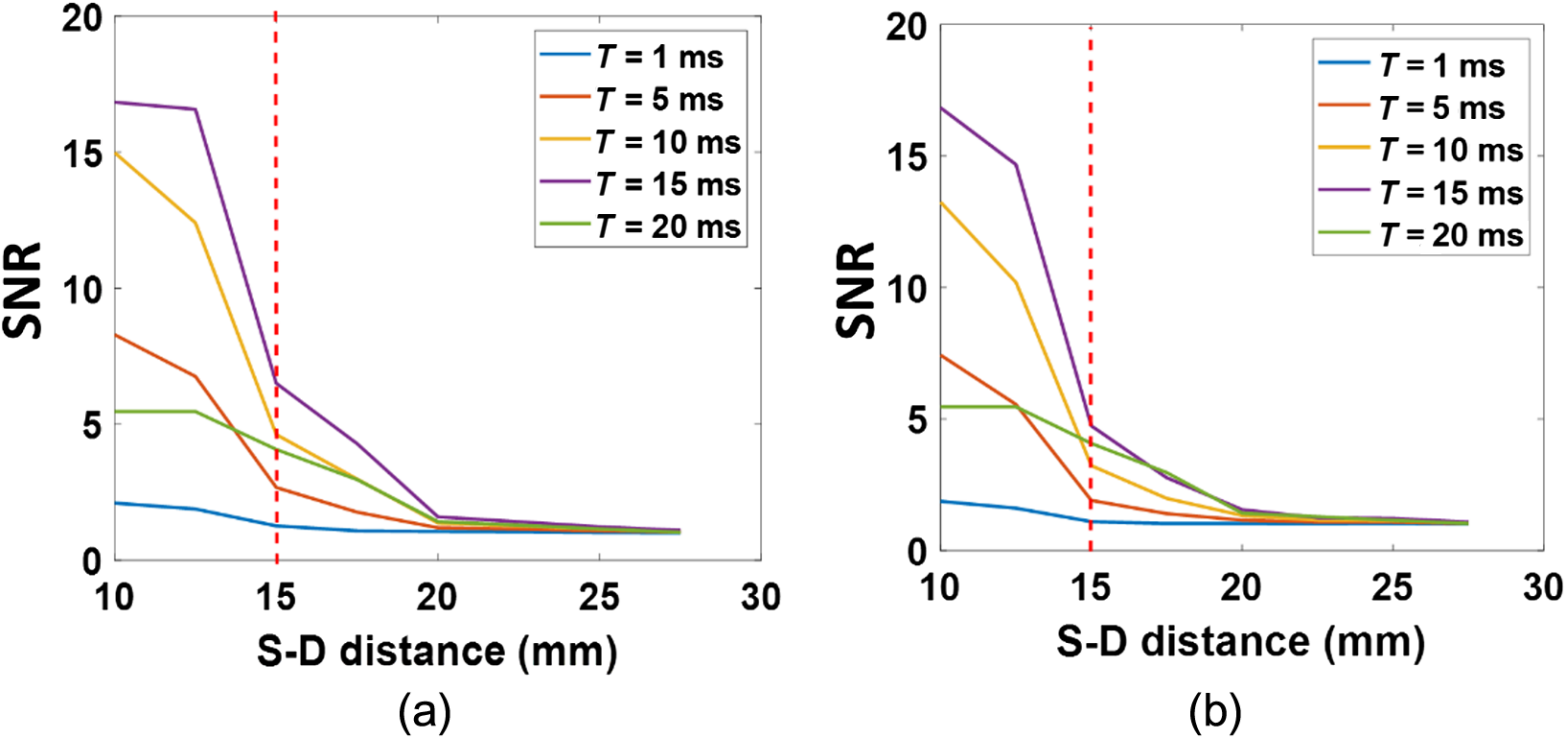
SNRs at different S-D distances with varied exposure times (T) measured in a standard tissue phantom ($\mu_{a}=0.025\;{\mathrm{cm}}^{-1}$ and ${\mu_{s}}^{\prime }=8\;{\mathrm{cm}}^{-1}$). (a) SNRs at 780 nm and (b) SNRs at 850 nm.

Figure 5 shows phantom measurement results to test the sensitivities and SNRs of the DSCFO at different S-D distances with varied exposure times for both wavelengths (780 and 850 nm). SNRs decreased with the increase of S-D distance and decrease of the exposure time for both wavelengths [Figs. 5(a) and 5(b)]. These results are expected as larger S-D distances and shorter exposure times resulted in fewer photons being detected, thus leading to lower SNRs.

**Fig. 6:**
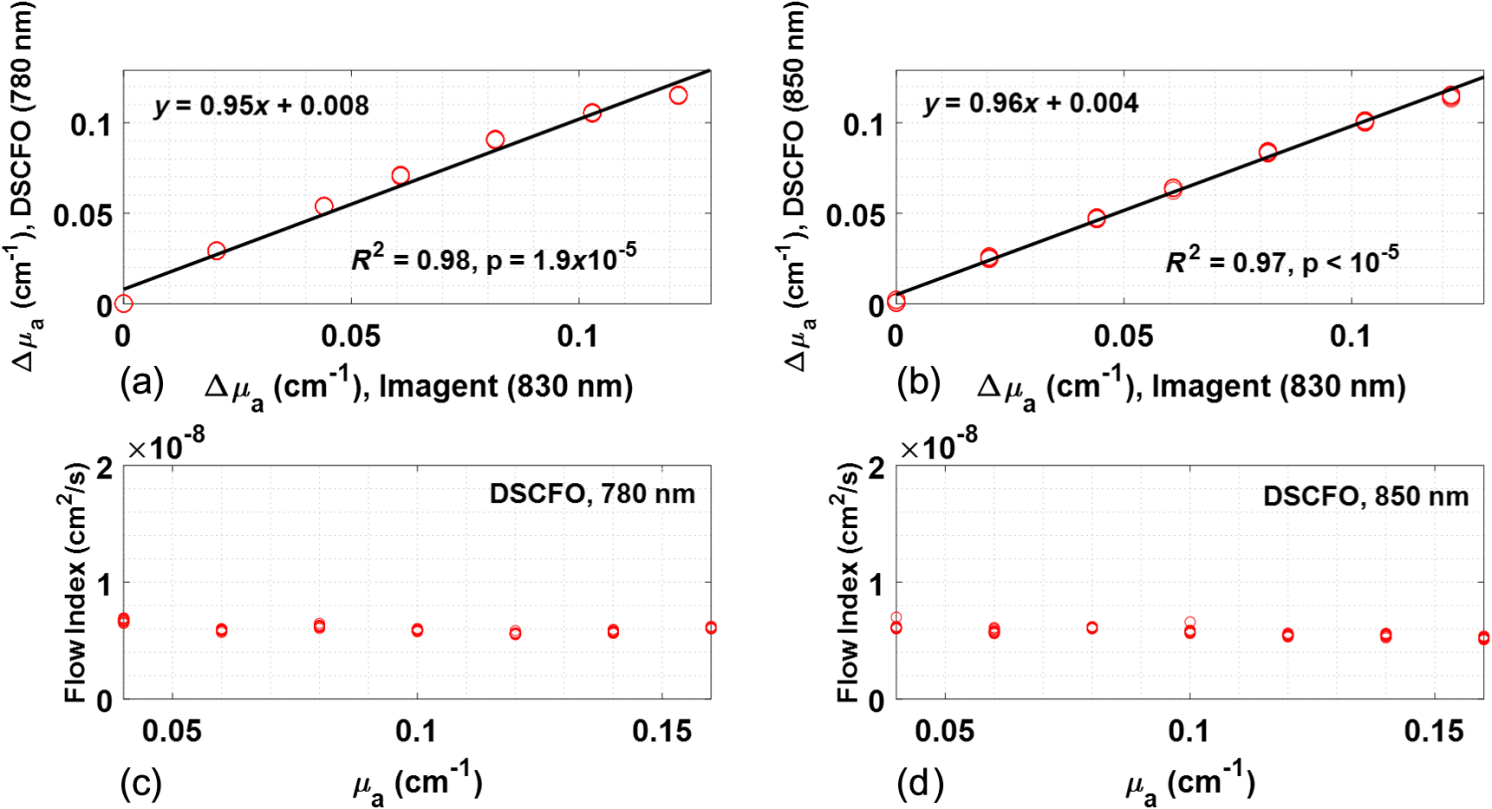
Phantom test results measured concurrently by the DSCFO and Imagent. (a) and (b) Significant correlations between the Imagent (at 830 nm) and DSCFO measurements of $\Delta \mu_{a}$ during ink titrations at two wavelengths (780 and 850 nm), respectively. (c) and (d) Flow index variations during ink titrations measured by the DSCFO at two wavelengths (780 and 850 nm), respectively. The camera exposure time was 10 ms for DSCFO measurements. The S-D distances for DSCFO and Imagent measurements were 15 and 20 mm, respectively. Twenty data points (10 for each wavelength) at each titration step were collected by the DSCFO with a sampling rate of 2 Hz.

**Fig. 7:**
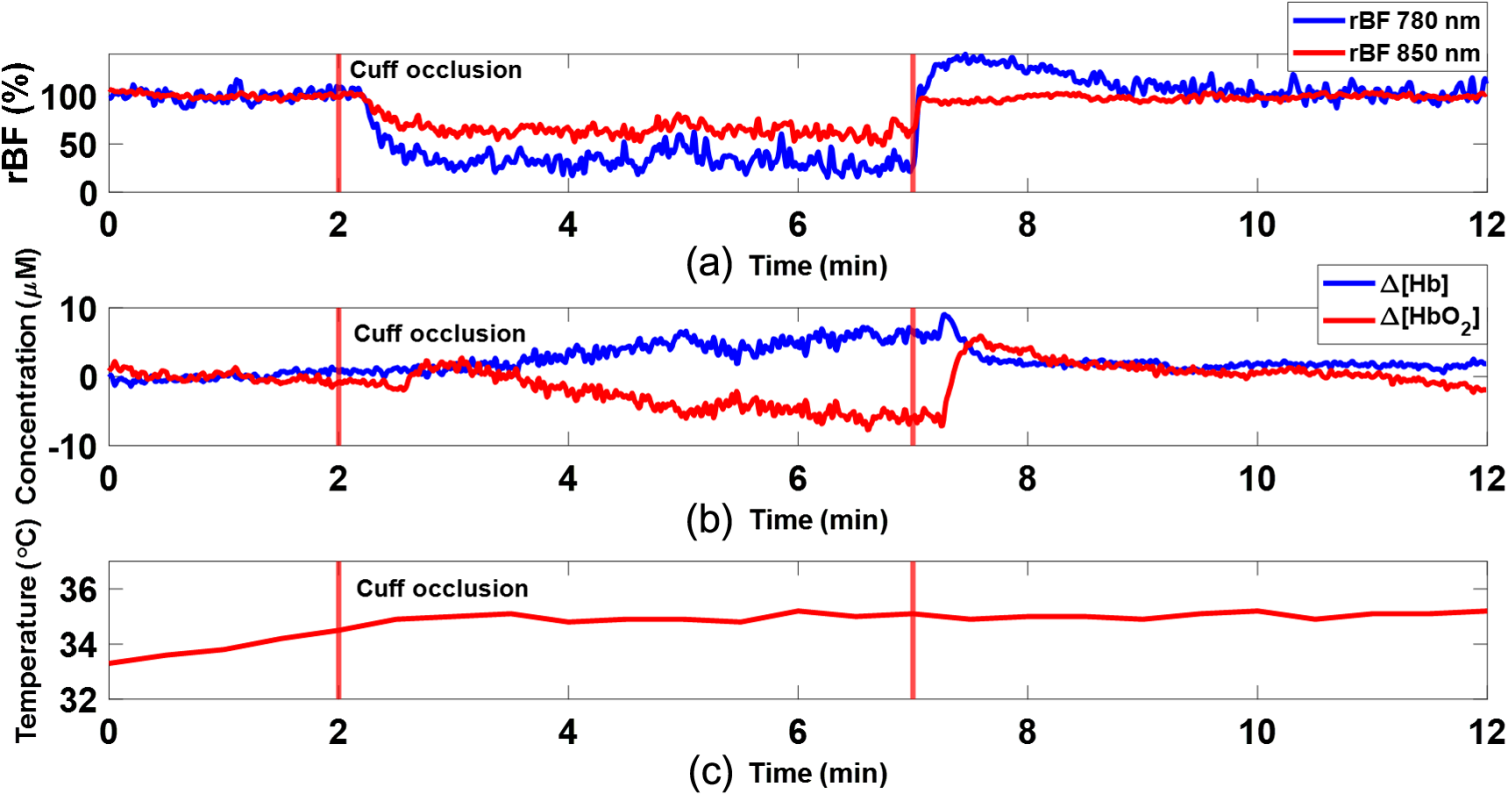
Typical hemodynamic changes during artery cuff occlusion measured by the DSCFO in one subject. (a) rBF responses to artery cuff occlusion induced by an inflation pressure of 220 mmHg. (b) Δ[Hb] and $\Delta [{\mathrm{HbO}}_{2}]$ responses to artery cuff occlusion. The experimental protocol included a 2-min baseline, a 5-min cuff-occlusion, and a 5-min recovery. (c) Continuous temperature variation on skin surface throughout the experimental period of 12 min.

**Fig. 8:**
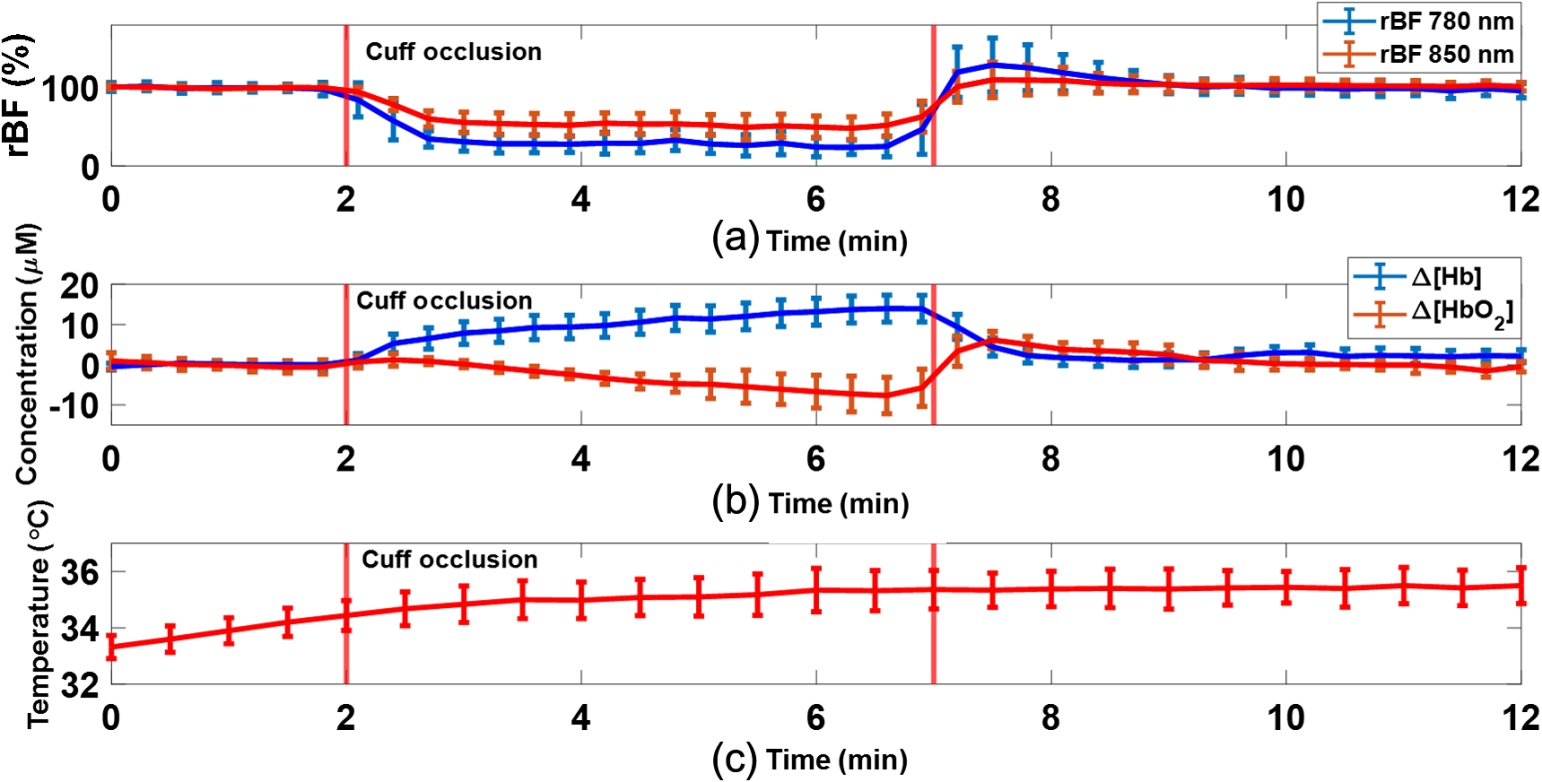
Average hemodynamic changes during artery cuff occlusion measured by the DSCFO over five subjects. (a) Average rBF responses to artery occlusion induced by an inflation pressure of 220 mmHg. (b) Average Δ[Hb] and $\Delta [{\mathrm{HbO}}_{2}]$ responses to artery cuff occlusion. (c) Temperature variations on skin surface during experiments. The error bars represented the standard deviations over five subjects.

However, when the exposure time increased continually and reached 20 ms, SNRs decreased due to the limited dynamic range of the CMOS sensor used in our DSCFO device. Furthermore, previous studies found that speckle contrast noises increased with the increase of exposure time.^58^^,^^59^ Thus, the contrast-to-noise ratio decreased when the exposure time was greater than 5 ms.^58^ Accordingly, the S-D distance of 15 mm and exposure time of 10 ms were used in following tests (Figs. 6–8) to optimize the light penetration depth and SNRs.

Figure 6 shows the comparison results in $\Delta \mu_{a}$ during ink titrations in tissue phantoms, measured concurrently by the DSCFO and Imagent devices. The wavelength of 830 nm in the Imagent was selected for comparisons as it is closer (than the 690 nm) to the two wavelengths used in the DSCFO (780 and 850 nm). Significant correlations were observed between the two measurements at two wavelengths (780 nm: regression slope = 0.95, $R^{2}=0.98$, $p\text{-value}=1.9\times 10^{-5}$; 850 nm: regression slope = 0.96, $R^{2}=0.97$, $p\text{-value}<10^{-5}$), verifying the capability of DSCFO in quantification of $\Delta \mu_{a}$ [Figs. 6(a) and 6(b)]. As expected, flow values measured by both wavelengths of DSCFO were fairly constant (maximum variations <6%) during ink titrations and discrepancies between the two measurements were <5% [Figs. 6(c) and 6(d)].

### 3.2 *In-Vivo* Blood Flow and Oxygenation Responses during Artery Cuff Occlusion

Figure 7(a) shows the typical response of rBF in the forearm to artery cuff occlusion (220 mmHg) in one subject measured by the DSCFO at two wavelengths (780 and 850 nm). The time-course variations in rBF measured at the two wavelengths showed a consistent dynamic trend although the magnitudes of rBF were somewhat different. Artery occlusion caused a significant reduction in rBF while the release of occlusion resulted in an immediate hyperemic response (rBF overshot), followed by a graduate recovery to the baseline.

Figure 7(b) shows typical dynamic changes in Δ[Hb] and $\Delta [{\mathrm{HbO}}_{2}]$ during artery cuff occlusion measured by the DSCFO in the same subject. During artery occlusion, Δ[Hb] increased while $\Delta [{\mathrm{HbO}}_{2}]$ decreased. After releasing occlusion, Δ[Hb] and $\Delta [{\mathrm{HbO}}_{2}]$ recovered gradually to their baselines.

Figure 7(c) shows the time-course temperature change on skin surface throughout the measurement period of 12 min. After an initial increase of ∼2°C within the first 2 to 3 min, skin surface temperature reached a plateau.

Figure 8(a) shows average dynamic changes in rBF during artery occlusion over five subjects, measured by the DSCFO at two wavelengths (780 and 850 nm). Time-course results are presented as the mean values ± standard deviation (error bars). Thirty data points were averaged and plotted with corresponding error bars to improve the readability. Similar trends in rBF were observed in the 2-wavelength measurements although the magnitudes of rBF differed slightly. rBF values during artery occlusion dropped to 0.15±0.05 (780 nm) and 0.41±0.14 (850 nm), respectively, from the baseline value of “1.” In particular, rBF at 780 nm dropped to 0.15±0.05, which is similar to the minimal value of 0.13 during artery occlusion, reported in our previous study using a single-wavelength DSCF at 780 nm.^21^

Figure 8(b) shows average changes in Δ[Hb] and $\Delta [{\mathrm{HbO}}_{2}]$ during artery occlusion over five subjects, measured by the DSCFO. The occlusions resulted in an increase of 15.1±3.2  μM in Δ[Hb] and a decrease of 8.3±4.7  μM in $\Delta [{\mathrm{HbO}}_{2}]$. After releasing occlusion, Δ[Hb] and $\Delta [{\mathrm{HbO}}_{2}]$ recovered gradually to their baselines. These dynamic changes are similar to those reported in previous studies using similar artery occlusion paradigms and NIRS devices.^25^^,^^26^^,^^54^^,^^60^

Figure 8(c) shows the average temperature change on skin surface during DSCFO measurements over five subjects. There was a rapid temperature increase of 2.2±0.4°C on skin surface within the first 2 to 3 min, followed by a stable temperature below the body temperature of 37°C. No subject reported any heat or uncomfortable feeling from the forearm during the DSCFO measurements. The initial temperature increase was likely attributed to slight heat accumulation on skin surface underneath the DSCFO probe. A new balance between the heat accumulation and spreading was rapidly established within 2 to 3 min after installing the DSCFO probe, leading to a stable skin surface temperature.

## 4 Discussion and Conclusions

Since multiple hemodynamic parameters provide more comprehensive assessment of tissue health than a single parameter alone, we extended our innovative single-wavelength DSCF^21^^,^^43^ (measuring blood flow alone) to a dual-wavelength DSCFO for simultaneous measurements of blood flow and oxygenation in deep tissues (Fig. 1). DSCFO uses small NIR laser diodes at different wavelengths as focused point sources for deep tissue penetration and a tiny CMOS sensor as a 2D detector array to detect spatial dynamic light scattering by intrinsic motions of red blood cells (i.e., blood flow) and light attenuations by oxy-hemoglobin and deoxy-hemoglobin absorptions ($\left[{\mathrm{HbO}}_{2}\right]$ and [Hb]). The unique DSCFO technology includes a portable device and a miniaturized fiber-free optical probe that can be worn by the subject for continuous monitoring of tissue hemodynamic variations. Importantly, connections between the probe and device are all electrical wires/cables (i.e., fiber-free), thereby offering the promise for continuous monitoring in freely behaving subjects.

We used 3D printing to fabricate the wearable DSCFO probe that bound two small laser diodes as dual-wavelength sources and a tiny CMOS camera as a 2D detector array [Fig. 1(e)]. The 3D fabricated probe allows easy installation of opto-electronic elements and precise fixation of S-D distance. Importantly, the unique probe design enables dissipating the heat generated by the laser diodes to prevent skin injury, thus allowing for continuous and longitudinal measurements. Moreover, the thermal sensor in the DSCFO probe continuously monitors skin surface temperature, which may be used as feedback control to ensure skin safety. For example, the DSCFO measurement may be terminated automatically when the skin temperature exceeds a safety threshold.

Tissue-simulating phantoms with known optical properties are commonly used to calibrate diffuse optical instruments.^44^^,^^55^ Following our established experimental protocols,^21^^,^^26^^,^^43^^,^^54^ the new DSCFO system was first characterized in a standard homogenous tissue phantom to determine the effective S-D distance and optimal exposure time for maximizing SNRs (Fig. 2). With the laser diodes and camera used in this DSCFO, the largest effective S-D distance was 15 mm and the optimal exposure time was 10 ms (Fig. 5). The S-D distance of 15 mm allowed for probing tissues at a maximal depth of ∼8  mm (1/2 of the S-D distance).^43^

An ink titration phantom test was then conducted against a commercial NIRS device (Imagent) to validate the accuracy in measuring $\Delta \mu_{a}$ (Fig. 3). The excellent correlation between the two measurements (DSCFO and Imagent) verified the capability of DSCFO in measuring $\Delta \mu_{a}$ (Fig. 6). Our previous study also verified the accuracy of single-wavelength DSCF (using a laser diode at 780 nm and the same NanEye camera) against the established DCS in measuring dynamic flow changes.^21^

After the phantom tests, the DSCFO was further examined for continuous monitoring of blood flow and oxygenation variations in human forearms during artery cuff occlusion (Fig. 4). The artery occlusion resulted in substantial changes in rBF, Δ[Hb], and $\Delta [{\mathrm{HbO}}_{2}]$ (Figs. 7 and 8), which meet physiological expectations and are consistent with previous study results using similar experimental protocols and technologies.^21^^,^^26^^,^^43^^,^^54^^,^^60^ Moreover, continuous monitoring of temperature variations on skin surface verified the safety of DSCFO measurements.

We recognize challenges and limitations in this pilot study and consider future improvements. Two laser diodes (D780-30: Ø5.6  mm, 30 mW, 780 nm, US-Lasers; L850P030: Ø5.6 mm, 30 mW, 850 nm, Thorlabs) were used as light sources in this study. These laser diodes oscillate in a single transverse mode, but support multiple longitudinal modes and would not be expected to exhibit a long-coherence length. However, few mode lasers have complex correlation functions that are not well described by a single coherence length. We previously placed a laser diode (L785P25, Ø5.6  mm, 25 mW, 780 nm, Thorlabs) in a Michaelson interferometer and operated it under the same conditions as in the DSCFO. High-visibility (>0.8) fringes were observed at many path-length differences between 0 and 400 mm (limit of test).^43^ Thus, the laser’s coherence appears entirely sufficient for the S-D separations investigated in this study.

Ideally, our dual-wavelength DSCFO with the NanEye camera at the highest frame rate of 50 Hz can achieve a sampling rate of 25 Hz. Practically, the maximal sampling rate of DSCFO is lower than 25 Hz because of the switching time between the two laser diodes and the delay time for stabilizing laser intensity outputs. In our previous study, we have demonstrated the capability of DSCF with a sampling rate of 20 Hz to capture the pulsatile blood flow.^21^ With such a high sampling rate, we can quantify laser speckle contrasts using both temporal and spatial calculations. We selected spatial calculations in this study as they generated higher sampling rates than temporal calculations. In the future, we may explore temporal calculations to improve the spatial resolution. Moreover, time-course data can be averaged to improve the SNR. To balance the sampling rate and SNR in this study, we chose 2 Hz to capture relatively slow changes in tissue hemodynamics induced by the artery occlusion.

We notice magnitude discrepancies in rBF responses to artery occlusions measured by the two wavelengths (Figs. 7 and 8), which are partially due to different spectral sensitivities of NanEye camera: 83% at 780 nm and 51% at 850 nm.^61^ A camera with higher sensitivity and quantum efficiency, larger bit level, and lower shot/dark noises would increase detection dynamic range. Moreover, 780 nm is not the optimal wavelength for blood oxygenation measurements as it is close to the isosbestic point.^62^ However, we have previously validated a dual-wavelength (785 and 854 nm) DCS system for simultaneous measurements of tissue blood flow and oxygenation variations.^26^^,^^54^ Similarly, the selection of two wavelengths in this study depended on availability of laser diodes in the laboratory. Although 780 and 850 nm may not be the optimal wavelengths for blood oxygenation measurements, the present study verified the feasibility of DSCFO with these two wavelengths to detect variations in $\left[{\mathrm{HbO}}_{2}\right]$ and [Hb]. Optimization of wavelengths for better separation of $\left[{\mathrm{HbO}}_{2}\right]$ and [Hb] is the object of our future study.

As a wearable device, motion artifacts could be a major concern when applying DSCFO measurements on freely behaving subjects. We can learn from previous studies with wearable NIRS techniques to optimize the probe design and fixation of the probe to minimize motion artifacts.^31^^,^^36^^,^^37^ For repeated measurements, mispositioning of the probe may affect the measurement repeatability. However, increasing the sampling rate or using temporal speckle contrast calculations would not solve this issue as mispositioning errors result primarily from tissue response heterogeneities at different locations. Ultimately, we expect to affix a wearable DSCFO probe on the same location of target tissues to avoid probe mispositioning.

Our wearable DSCFO technique allows noninvasive measurements of both blood flow and oxygenation in relatively deep tissues (up to ∼10  mm). Supported by the National Institutes of Health pilot grants, we are currently optimizing this technique for noninvasive cerebral monitoring in rodents, piglets, and human neonates. We will optimize the sources (e.g., wavelengths and intensities) and detectors (e.g., better quality cameras) to further improve measurement sensitivity, SNR, and penetration depth, with the goal of measuring cerebral hemodynamics in human adults. Since parts/components used to build the reusable DSCFO probe are relatively inexpensive, we can integrate multiple laser diodes and cameras into larger probes (such as conventional NIRS/DCS probes^63^^–^^67^) to cover larger tissue volumes and improve sampling density. Moreover, the DSCFO controller (Arduino Uno board) has multiple I/O channels to control multiple modules and probes.

We will explore wireless data transfer (via Bluetooth or Wi-Fi on the Arduino Uno board) to develop a battery powered wearable DSCFO device for continuous and simultaneous monitoring of blood flow and oxygenation in freely behaving subjects. We will also conduct concurrent *in-vivo* measurements with other established techniques such as NIRS and DCS to further verify the accuracy and reproducibility of DSCFO measurements in large populations. Ultimately, we expect to offer a noninvasive, inexpensive, and wearable device for continuous monitoring of tissue hemodynamics to investigate pathologies and interventions for various vascular/cellular diseases.
